# Identification of the Major Facilitator Superfamily Efflux Pump KpsrMFS in *Klebsiella pneumoniae* That Is Down-Regulated in the Presence of Multi-Stress Factors

**DOI:** 10.3390/ijms25031466

**Published:** 2024-01-25

**Authors:** Wei He, Minzhi Jiang, Ying Li, Xizhen Ge

**Affiliations:** College of Biochemical Engineering, Beijing Union University, Beijing 100023, China

**Keywords:** efflux pump, antibiotic resistance, *Klebsiella pneumoniae*, down-regulated, reactive oxygen species

## Abstract

Efflux pumps play important roles in bacterial detoxification and some of them are stress-response elements that are up-regulated when the host is treated with antibiotics. However, efflux pumps that are down-regulated by stimulations are rarely discovered. Herein, we analyzed multiple transcriptome data and discovered a special (Major Facilitator Superfamily) MFS efflux pump, KpsrMFS, from *Klebsiella pneumoniae*, which was down-regulated when treated with antibiotics or extra carbon sources. Interestingly, overexpression of *kpsrmfs* resulted in halted cell growth in normal conditions, while the viable cells were rarely affected. The function of KpsrMFS was further analyzed and this efflux pump was determined to be a proton-driven transporter that can reduce the intracellular tetracycline concentration. In normal conditions, the expression of *kpsrmfs* was at a low level, while artificial overexpression of it led to increased endogenous reactive oxygen species (ROS) production. Moreover, by comparing the functions of adjacent genes of *kpsrmfs*, we further discovered another four genes that can confer similar phenotypes, indicating a special regulon that regulates cell growth. Our work provides new insights into the roles of efflux pumps and suggests a possible regulon that may regulate cell growth and endogenous ROS levels.

## 1. Introduction

*Klebsiella pneumoniae* can cause a wide range of infections including pneumonia, urinary tract infections, bacteremia, and liver abscesses in people who are immunocompromised [1]. However, the recent emergence and spread of hypervirulent strains have led to infections in individuals who are both healthy and immunosufficient [2,3], presenting significant risks to global health [4]. Among the elements that can facilitate cell survival in the presence of antibiotics, an efflux pump is an important one that can export toxins out of the cell cytoplasm [5]. With rapid evolution and horizontal transfer of efflux pumps, they are becoming the leading cause of multi-drug resistance across different bacterial species [6,7].

Bacterial efflux pumps also play an essential role in cell metabolism [5]. Up to now, efflux pumps have been demonstrated to affect various cell functions, such as maintaining a healthy cell envelope, forming biofilm, sustaining homeostasis, and regulating virulence [8,9,10,11]. More importantly, bacteria can use these efflux pumps to defend against harmful substances that threaten their survival. These harmful substances include not only internal toxic metabolites but also external antibiotics [12]. In bacteria, seven families of efflux pumps have been discovered that can lead to multi-drug resistance, namely the ATP-binding cassette (ABC) family, the resistance/nodulation/cell division (RND) family, the multi-drug and toxic compound extrusion (MATE) family, the major facilitator superfamily (MFS), the small multi-drug resistance (SMR) family, the proteobacterial antimicrobial compound efflux (PACE) family, and the p-aminobenzoyl-glutamate transporter (AbgT) family [13]. Efflux pumps in different families have distinct roles and substrates in transporting antibiotics out of the cytoplasm, leading to the failure of antibiotic therapy. Therefore, investigating the function, evolution, and transfer of efflux pumps is crucial for the treatment of bacterial infections [6,14].

Efflux pumps are membrane proteins of bacteria. For Gram-negative bacteria, monomer efflux pumps are embedded in the inner membrane, while the tripartite RND efflux pump is located across the inner and outer membrane [15]. In normal conditions without stimulation, some efflux pumps are constitutively expressed to transport secondary metabolites out of the cytoplasm, while others are silent [16,17,18]. The expression levels of efflux pumps are crucial for cell metabolism and homeostasis, and either overexpression or inhibition of efflux pumps can cause fitness defects [19,20]. However, with stimulation caused by antibiotics or other toxins, the expression levels of several efflux pumps are up-regulated for the accelerated export of harmful compounds. These stress-response efflux pumps have been demonstrated to play a crucial role in cell survival under the treatment of antibiotics, such as the SMR efflux pump KpnEF in *Klebsiella pneumoniae* and the RND efflux pump OqxAB in *Escherichia coli* [21,22]. Indeed, bacteria utilize numerous stress response mechanisms to regulate the expression levels of efflux pumps precisely for adaptation to the environment [23,24]. However, in *K. pneumoniae*, numerous efflux pumps are distributed widely on the chromosome and plasmids [25]. The distinct roles of these efflux pumps in regulating cell growth or cell functions need to be further investigated.

With the decreased cost of Next Generation Sequencing, more and more transcriptome profiles are obtained under different conditions, providing sufficient data for the investigation of genes that correspond to stress. Herein, we analyzed 309 transcriptome samples of *K. pneumoniae* from our experiments and SRA data of NCBI to identify stress-response efflux pumps. By comparing the transcriptome data of *K. pneumoniae* under normal conditions (in MHB medium), we unexpectedly discovered a special MFS efflux pump KpsrMFS that was down-regulated in the presence of different antibiotics or extra carbon sources. We used molecular biology approaches to investigate the role of KpsrMFS. Interestingly, we found that overexpression of *kpsrmfs* resulted in halted cell growth while the viable cells were rarely affected. Moreover, we discovered four adjacent genes of *kpsrmfs* that resulted in similar phenotypes when individually overexpressed in *K. pneumoniae*, suggesting these genes may constitute a special regulon in response to stresses. We further confirmed the halted cell growth was related to elevated endogenous ROS levels. Our study revealed a special efflux pump and suggests a potential regulon that can affect endogenous ROS levels.

## 2. Results

### 2.1. Identification of Genes That Had the Largest Fluctuations in Different Conditions

**Figure 1:**
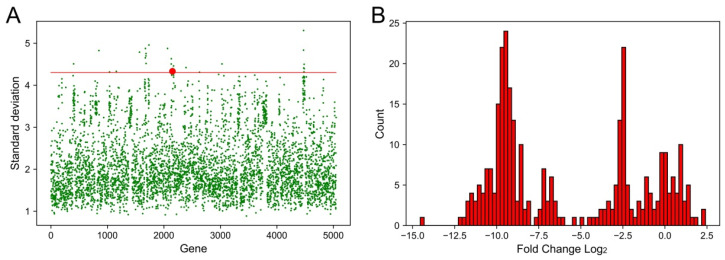
Identification of an efflux pump KpsrMFS that has the largest variation in transcriptome profiles of *K. pneumoniae*. Transcriptome profiles of *K. pneumoniae* under different conditions (half of the MIC values of *K. pneumoniae* in our work, Supplementary Materials, Table S1) were analyzed and compared with the profiles of *K. pneumoniae* in MHB medium, and the standard deviation of each gene was used to assess the fluctuation. (**A**) Fluctuation of each gene in *K. pneumoniae* through transcriptome analyses. Spots above the red line indicate the top 25 genes that had the largest fluctuations and the red spot indicates the MFS efflux pump KpsrMFS. (**B**) Distribution of the fold changes in *kpsrmfs* under different conditions.

In order to identify genes that might be stress-responsive, we analyzed transcriptome profiles of *K. pneumoniae* under different conditions (half of the MIC values of *K. pneumoniae* in this work, Supplementary Materials, Table S1) and calculated the fluctuation of each gene. By comparing the profiles of *K. pneumoniae* in the MHB medium, we selected the top 25 genes that had the largest standard deviations (Table 1 and Figure 1A). Among these genes, a special MFS efflux pump was suspected to contribute to multi-drug resistance since some other stress response efflux pumps had been identified in *K. pneumoniae* [21]. This MFS efflux pump was then named KpsrMFS and the coding gene was designated as *kpsrmfs*. Next, we analyzed the detailed changes in *kpsrmfs* and obtained the histogram (Figure 1B). Unexpectedly, *kpsrmfs* was down-regulated under most of the conditions, while no significant up-regulation was detected in all these samples. The fold changes in *kpsrmfs* were mainly distributed around three areas. The first area was around 0 because these samples contained control experiments when *K. pneumoniae* was cultivated in LB or MHB medium without antibiotics. In comparison, the other two areas were located around −2.5 and −10, which indicates that *kpsrmfs* tended to be down-regulated in different conditions.

### 2.2. Overexpression of Kpsrmfs Resulted in Halted Cell Growth

**Figure 2:**
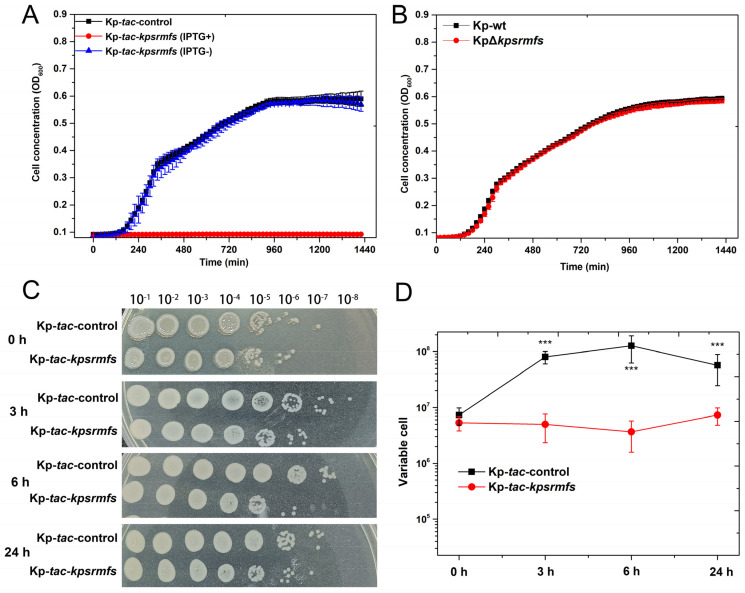
Overexpression of *kpsrmfs* conferred halted cell growth of *K. pneumoniae*, while the viable cells were not reduced. Growth curves of the recombinant strains were conducted in a 96-well plate with IPTG supplied, and killing assay-like assays were carried out via 10-fold dilution of the overnight culture in fresh medium with IPTG added. (**A**) Cell growth *K. pneumoniae* with *kpsrmfs overexpressed* was not observed within 24 h, while the overexpressor without IPTG displayed normal growth. The medium contained 50 μg/mL of chloramphenicol to sustain the plasmid. (**B**) Cell growth of *K. pneumoniae* with *kpsrmfs* knockout was not affected. (**C**,**D**) Killing assay-like assessment of Kp-*tac*-*kpsrmfs* with the inducer IPTG supplied in the medium. The experiments were biologically replicated three times and the asterisk indicates the *p*-value (***: *p* < 0.001).

To further identify the roles of *kpsrmfs* in regulating the phenotypes of *K. pneumoniae*, we overexpressed this gene on the plasmid with a tightly inducible *tac* promoter. Surprisingly, overexpression of *kpsrmfs* conferred severe growth defects within 24 h (Figure 2A), while the growth of this recombinant strain was rarely affected without IPTG supplied. At the same time, knockout of *kpsrmfs* had little effect on cell growth (Figure 2B). In parallel, we conducted killing assay-like experiments with IPTG and chloramphenicol supplied in fresh MHB culture, and the overnight culture of Kp-*tac*-*kpsrmfs* (without IPTG) was used and 10-fold diluted into the fresh medium for the assay (Figure 2C). In the overnight culture, the viable cells of Kp-*tac*-*kpsrmfs* and the vector control Kp-*tac*-control were similar (0 h). However, when *kpsrmfs* was induced for 3 h to the final 24 h (SDS-PAGE in Figure S1), the viable cells of Kp-*tac*-*kpsrmfs* remained the same as the initial. The results indicate that even though cell growth was halted, these cells were still alive when this special efflux pump was overexpressed. These data suggest that overexpression of *kpsrmfs* conferred halted growth of *K. pneumoniae* [26], while it was not lethal to the host.

In contrast, we tested the MIC values of *K. pneumoniae* wild type and the *kpsrmfs* mutant strains. Even though overexpression of *kpsrmfs* conferred halted cell growth, the deletion of this gene still resulted in significantly decreased resistance to ciprofloxacin, tetracycline, and azithromycin by >4-fold. The MIC data were somehow paradoxical to cell growth. These data imply that even though over-provided *kpsrmfs* restricted cell growth, it still played an important role in transporting toxins at their original expression level. At the same time, we constructed a strain Kp-pET-*Pstr*-*kpsrmfs* in which the original expression cassette (*kpsrmfs* and its native promoter *Pstr*) was assembled into the backbone of pET-28a that lacks the T7 promoter. With the increased copy number of the cassette, antibiotic susceptibilities of *K. pneumoniae* were not altered.

### 2.3. KpsrMFS Is an Active Efflux Pump for Tetracycline Export

**Figure 3:**
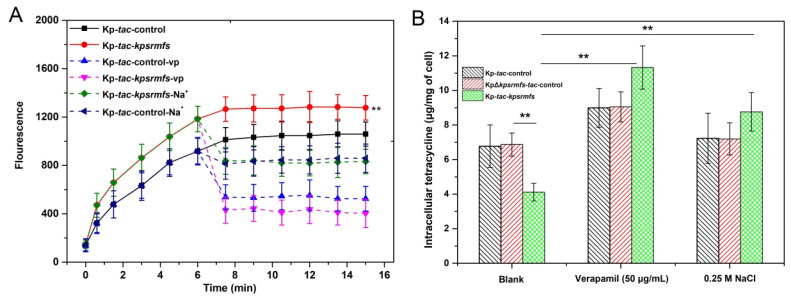
KpsrMFS can export tetracycline when overexpressed on plasmid. Recombinant cells were cultivated overnight without IPTG for cell growth, and then *kpsrmfs* was induced by the addition of IPTG for 3 h (**A**) Tetracycline efflux assay of KpsrMFS with different inhibitors added. (**B**) Intracellular tetracycline concentrations of the recombinant *K. pneumoniae* strains cultivated with 100 μg/mL of tetracycline for 3 h. The arrow indicates the time point when the inhibitor or 0.25 M of NaCl was added. Vp was the MFS efflux pump inhibitor verapamil and the asterisk indicates the *p*-value of three biological replicates (**: *p* < 0.01).

Overexpression of *kpsrmfs* conferred halted cell growth, which indicates that the efflux pump should be induced when biomass reaches a certain level. In order to identify the role of KpsrMFS in exporting antibiotics, we supplied inducer IPTG and tetracycline after cell concentration was accumulated (Figure 3). In the tetracycline efflux assay, overexpression of *kpsrmfs* conferred a slightly increased export level compared with the control (Figure 3A), and inhibitor verapamil reduced fluorescence level when added to the reaction. Notably, when 0.25 M NaCl was provided in the reaction, the fluorescence level was also decreased. These data suggest that KpsrMFS can export tetracycline depending on the proton motive force [27]. In parallel, we determined intracellular tetracycline concentrations of these recombinant strains with inhibitors supplied (Figure 3B). Overexpression of *kpsrmfs* resulted in a decrease of nearly 40% in intracellular tetracycline concentration, while deletion of *kpsrmfs* had little impact on the antibiotic level. In contrast, the addition of verapamil led to increased intracellular tetracycline levels of all these strains, while NaCl only significantly impacted that of the *kpsrmfs* overexpressor. These results confirm that KpsrMFS can efflux tetracycline when induced in *K. pneumoniae*, and its active export may rely on proton motive force.

### 2.4. Kpsrmfs Was Constitutively Expressed but Down-Regulated in the Presence of Multi-Stress Factors

**Figure 4:**
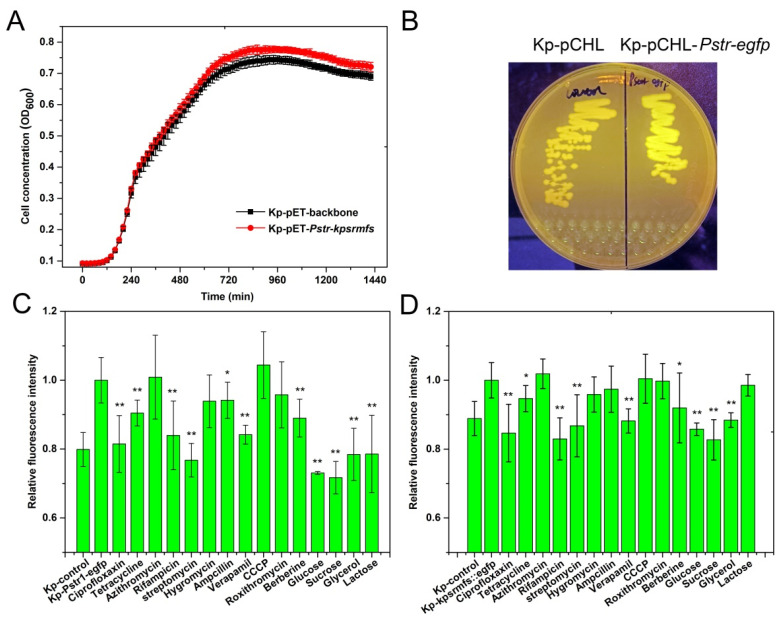
*Kpsrmfs* was constitutively expressed at a low level but was down-regulated in the presence of antibiotics or extra carbon sources. (**A**): The growth curve of *K. pneumoniae* with an increased copy number of the original expression cassette (*kpsrmfs* and its native promoter *Pstr*) on the plasmid was not affected. (**B**) Visualization of the reporter strain (Kp-pCHL-*Pstr*-*egfp*) with excitation at 488 nm. (**C**) Relative fluorescence levels of the reporter strain (Kp-pCHL-*Pstr*-*egfp*) under different conditions. (**D**) Relative fluorescence levels of the reporter strain (Kp-Δ*kpsrmfs*::*egfp*) under different conditions. The asterisk indicates the *p* value (*: *p* < 0.01; **: *p* < 0.01).

To reveal the transcription of *kpsrmfs* under different conditions, we first cloned the whole expression cassette containing the *kpsrmfs* gene and its native promoter *Pstr* into pET-28a, which lacks the T7 promoter. The copy number of the pET vector in bacteria was around 10 [28], and this means the amount of *Pstr*-*kpsrmfs* expression cassette was artificially enriched in *K. pneumoniae*. However, cell growth of the recombinant strain was rarely affected (Figure 4A), and the MIC values of this strain displayed a slight but not significant increase (Table 2). At the same time, we assembled a vector with the promoter *Pstr* and *egfp* gene to estimate its activation. Without stimulations, the *egfp* was constitutively expressed at a low level (Figure 4C). When different simulations were provided, the fluorescence levels were reduced by different degrees. Notably, in the presence of some carbon sources that can be used by *K. pneumoniae* [29], the expression levels of *Pstr* also decreased sharply, and their levels were even similar to the negative control without *egfp*. In parallel, we replaced the *kpsrmfs* gene with an *egfp* on the chromosome to generate another reporter strain. Similarly, the fluorescence levels of the reporter strain were moderately reduced in the presence of several antibiotics and extra carbon sources (Figure 4D).

### 2.5. Overexpression of Four Adjacent Genes of Kpsrmfs Resulted in Similar Phenotypes

**Figure 5:**
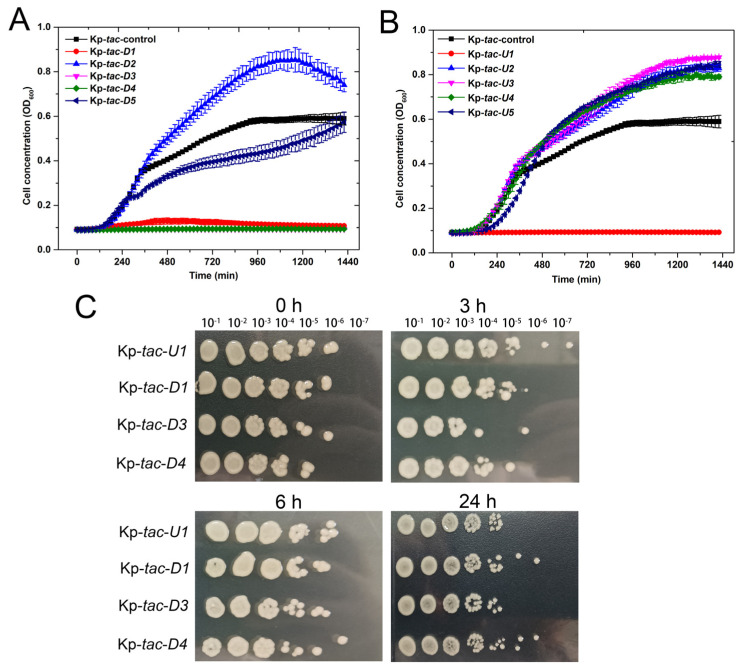
Overexpression of the adjacent genes of *kpsrmfs* resulted in similar phenotypes. (**A**,**B**) Growth curves of the recombinant strains that individually expressed upstream and downstream genes of *kpsrmfs*. A growth assay was conducted in a 96-well plate with IPTG supplied. (**C**) Viable cells of the recombinant strains with IPTG induced. Overnight cultures of these recombinant strains were diluted 10-fold and IPTG was then added to induce the expression of these genes. All of the experiments were carried out with three biological replicates.

**Figure 6:**
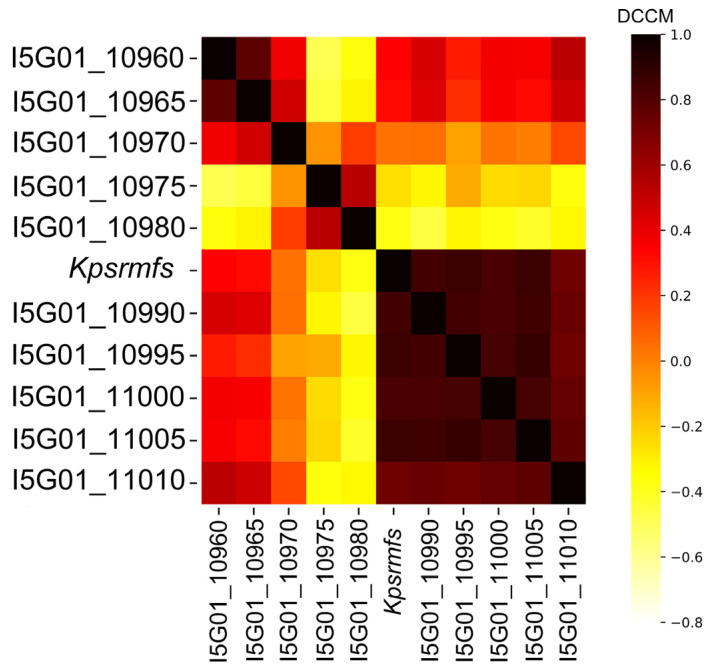
Dynamical Cross-Correlation Matrix (DCCM) of the adjacent gene expression level. Transcriptional synchronicity between each pair of genes was calculated and identified as positively related (0 to 1) or negatively related (−1 to 0).

Since we discovered a special MFS efflux pump in regulating the cell phenotype of *K. pneumoniae*, we checked whether this phenomenon can be reached by expressing adjacent genes or not. We selected the five upstream and downstream genes of *kpsrmfs* on the chromosome (Table 3) and overexpressed them individually on the same plasmid (Figure 5). Interestingly, another four genes were discovered to have similar impacts as *kpsrmfs*, and the growth of these four recombinant strains was strictly restricted within 24 h (Figure 5A). Similarly, inductions of these genes were not lethal to the host in the killing assay-like experiments (Figure 5B). Conversely, overexpression of several adjacent genes resulted in accelerated cell growth. Since some of these genes were also listed in the top 25 fluctuant ones (Table 1), we calculated their Dynamical Cross-Correlation Matrix (DCCM) to evaluate their synchronicities (Figure 6). The results indicate that the expression level of *kpsrmfs* is tightly correlated to its five downstream genes. However, synchronicity between *kpsrmfs* and its upstream gene *U1* was not observed although their overexpression resulted in similar phenotypes. Overall, the results suggest that these genes may constitute a special regulon in controlling cell phenotypes.

### 2.6. Overexpression of Kpsrmfs Conferred Slightly Reduced Tetracycline Tolerance in Killing Assay

**Figure 7:**
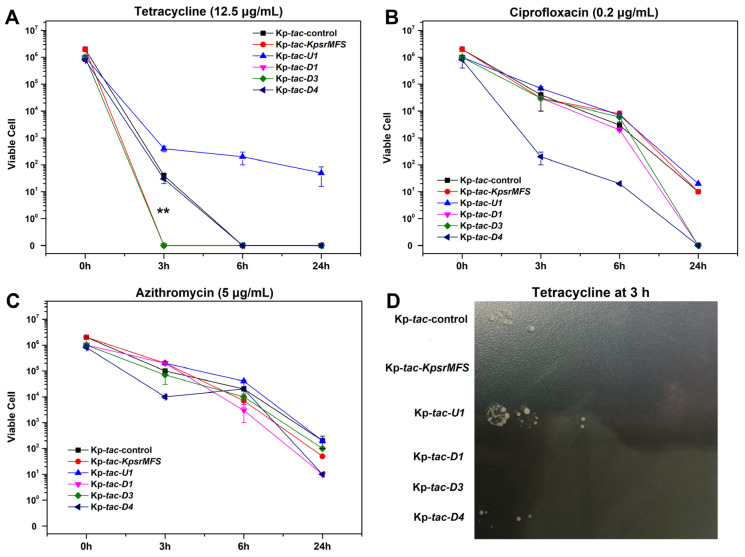
The recombinant Kp-*tac*-*kpsrmfs* displayed reduced tetracycline resistance in the killing assay. Overnight culture of the recombinant strains was diluted 10-fold in fresh medium with IPTG supplied. Next, different antibiotics were added and the viable cell was measured on a plate. (**A**–**C**) Viable cells of killing assay of tetracycline, ciprofloxacin, and azithromycin, respectively. (**D**) Bacterial plot of these recombinant strains treated with tetracycline for 3 h. All the experiments were biologically replicated. The asterisk indicates the *p*-value (**: *p* < 0.01).

Since overexpression of *kpsrmfs* resulted in halted cell growth, we conducted a killing assay to reveal the effects of these genes on antibiotic tolerance of tetracycline, ciprofloxacin, and azithromycin (Figure 7). With these genes induced in fresh medium, the major differences were observed when tetracycline was provided and reduced viable cells of Kp-*tac*-*kpsrmfs* and Kp-*tac*-*D3* were observed at 3 h. Notably, *D3* also encoded an MFS efflux pump in the deduced regulon. However, induced expression of the *U1* gene conferred increased tolerance to tetracycline from 3 h to the end of the experiment. On the other hand, the induced *D4* gene resulted in lower viable cells in ciprofloxacin, while these recombinant strains displayed little differences in azithromycin.

### 2.7. ROS Levels Were Significantly Elevated by Inducing Genes That Confer Halted Cell Growth

**Figure 8:**
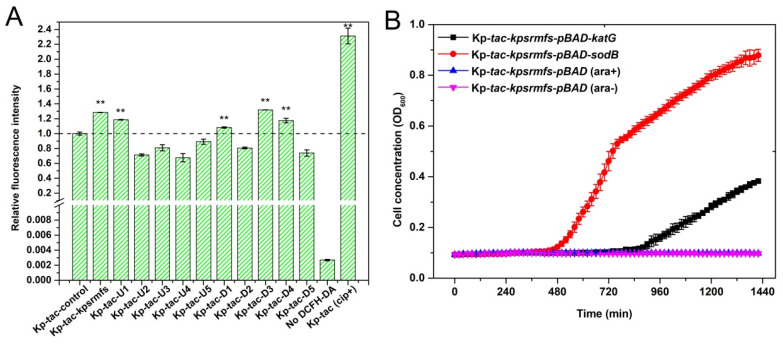
ROS levels were altered with different genes overexpressed in the deduced regulon, and complementation of catalase partially reversed cell growth. (**A**) Intracellular ROS levels of the recombinant strains overexpressing *kpsrmfs* and its adjacent genes. ROS probe DCFH-DA was used and the fluorescence was detected at excitation of 488 nm and emission of 525 nm. Cip+ indicates the cells were treated with 0.2 μg/mL of ciprofloxacin for 3 h, and this fluorescence intensity was used as the positive control. (**B**) Complemented overexpression of *katG* or *sdoB* partially restored cell growth. All the experiments were biologically repeated. The asterisk indicates the *p*-value (**: *p* < 0.01).

To further reveal the reason for halted cell growth, we determined the ROS levels of the recombinant strains with IPTG supplied. Unexpectedly, overexpression of genes that conferred halted cell growth resulted in significantly elevated ROS levels (Figure 8). Notably, the highest ROS levels were observed with overexpression of *kpsrmfs* and *D3*, two similar MFS efflux pumps in this special regulon of *K. pneumoniae*. Similarly, induction of *U1*, *D1,* and *D4* genes also promoted ROS levels by nearly 1.2-fold compared with the control. By using the ciprofloxacin-treated *K. pneumoniae* cells as the positive control, we discovered that the ROS levels of these recombinant strains were kept at an appropriate range. For the recombinant strain Kp-*tac*-*kpsrmfs*, the ROS level was nearly half of the positive control. On the contrary, overexpression of the other genes in this area led to reduced ROS levels, which may explain their accelerated cell growth (Figure 5). In summary, expression levels of these genes had special relationships with the ROS levels of the recombinant strains, which indicates that this special region on the chromosome may regulate cell metabolism by controlling endogenous ROS levels.

At the same time, we also explored the effect of catalase and superoxide dismutase on restoring cell growth when *kpsrmfs* was overexpressed. We used pBAD18-kan vectors to overexpress the native *katG* or *sodB* gene from *K. pneumoniae* together with *kpsrmfs* (Figure 8B). The catalase activity of recombinant *K. pneumoniae* when *katG* was overexpressed reached 126.5 U/mg protein, while the control group was only 45.3 U/mg. Similarly, SOD enzyme activity when *sodB* was overexpressed reached 79.3 U/mg protein, while the control group was only 22.3 U/mg. The results of growth curves indicate that the addition of both *katG* and *sodB* can partially restore cell growth when *kpsrmfs* was overexpressed, while the control strain Kp-*tac*-*kpsrmfs*-*pBAD* was unable to grow within 24 h. These data confirm that the halted cell growth of Kp-*tac*-*kpsrmfs* was affected by the increase in endogenous ROS levels to a proper range, in which the viable cell was not affected.

## 3. Discussion

Efflux pumps play a crucial role in regulating cell metabolism and mediating antibiotic resistance in bacteria [12]. Herein, we discovered the special MFS efflux pump, KpsrMFS, in *K. pneumoniae* that was down-regulated in the presence of multi-stress factors, and it was determined that overexpression of this efflux pump can confer halted cell growth. By assessing the viable cell and ROS levels of the recombinant strain, we revealed a novel role of efflux pumps in regulating the cell growth of *K. pneumoniae*. However, no homologs or paralogs of KpsrMFS were found in genera other than *Klebsiella* via NCBI blast analysis. This suggests that this particular efflux pump is exclusive to the *Klebsiella* genus.

Overexpression of efflux pumps has been used to investigate their roles in antibiotic resistance as well as in metabolic engineering [19,25]. In general, an over-provided efflux pump can disrupt homeostasis and reduce the fitness of the recombinant cell [30,31]. Conversely, overexpression of efflux pumps facilitates cell growth in harsh conditions [25]. In this circumstance, efflux pumps can export both exogenous and endogenous toxins, such as antibiotics and secondary metabolites [32]. However, for *kpsrmfs* identified in our work, overexpression of it confers significantly elevated ROS levels, while the generated ROS is not lethal to the cell and has little effect on the viability of *K. pneumoniae*. In the MHB medium, *kpsrmfs* is constitutively expressed, while it is down-regulated when antibiotics or other carbon sources are supplied. These data imply that there is a tight correlation between cell growth, *kpsrmfs* expression, and ROS level. Interestingly, the adjacent genes also have these special relationships with ROS levels, and these data are in agreement with their growth curves. Since most of these genes are synchronously regulated in DCCM analyses (Figure 6), these genes may constitute a special regulon in response to stresses, affecting cell growth and homeostasis [33,34].

In the deduced regulon, overexpression of the five genes confers halted cell growth, including two MFS efflux pumps (encoded by *kpsrmfs* and *D3*), a DUF35 family protein (encoded by *U1*), a helix-turn-helix transcriptional regulator (encoded by *D1*) and a chromate resistance protein (encoded by *D4*). Among these genes, the DUF35 (*U1*) is not positively correlated with the other ones under different conditions. Indeed, the DUF35 family protein is an important element involved in steroid metabolism and has been identified in oxidative stress [35]. Steroid metabolites are more than nutrients for pathogenic bacteria, and they may also function in other pathways contributing to bacterial virulence such as stress resistance [36]. This is in accordance with our data that overexpression of *U1* leads to increased tolerance to tetracycline in the killing assay (Figure 7). Therefore, these pathways and associated enzymes have received attention as potential targets for the development of new antibiotics [37]. In parallel, the helix-turn-helix transcriptional regulator (*D1*) is also reported as a sensor for oxidative stress in *Pseudomonas aeruginosa* and *Xanthomonas campestris* [38,39]. These regulators are DNA-binding proteins for sensing exogenous ROS when infected, and they can regulate genes involved in resistance to numerous toxins. However, these deductions seem paradoxical to the data that overexpression of this regulator increases endogenous ROS levels and confers halted cell growth. One possible explanation is that the regulator controls the expression of its adjacent MFS efflux pumps and the chromate resistance protein. Since overexpression of efflux pumps may disrupt cell homeostasis and result in growth defects [24], endogenous ROS levels will be elevated. In the medium without antibiotics, the KpsrMFS efflux pump is constitutively but weakly expressed, and the lack of it confers increased tetracycline susceptibility. This indicates that an appropriate expression level of *kpsrmfs* is essential for normal metabolism and cell growth. On the contrary, it is down-regulated in harsh conditions, which implies a proactive mechanism for cell survival and reduced endogenous ROS levels. Therefore, further biochemical experiments may be required to investigate the roles of this potential regulon in the formation of persisters when treated with antibiotics.

In summary, our data identified an MFS efflux pump that significantly affects the cell growth of *K. pneumoniae* and further discovered a deduced regulon that controls the endogenous ROS levels. We provided novel insights into the roles of the efflux pump in regulating cell growth and ROS levels.

## 4. Materials and Methods

### 4.1. Chemicals and Strains

*K. pneumoniae* was previously isolated from the biogas slurry residue, and ampicillin (10 μg/mL) was used in the following steps to separate this strain. The genome sequencing of this strain was completed and the genomic information is available from NCBI (Accession: ASM1583203v1) [40]. Tetracycline (Macklin, Shanghai, China), streptomycin (Sigma, Shanghai, China), hygromycin (Macklin, Shanghai, China), kanamycin (Macklin, Shanghai, China), and roxithromycin (Macklin, Shanghai, China) were formulated as a 2 mg/mL stock solution in distilled water and stored at 4 °C. Rifampicin (Macklin, Shanghai, China) was formulated as a 2 mg/mL stock in DMSO, and azithromycin (Sigma, Shanghai, China) and chloramphenicol (Macklin, Shanghai, China) were formulated as a 2 mg/mL stock solution in ethanol. Ciprofloxacin (Macklin, Shanghai, China) was dissolved in 0.15 M of sodium hydroxide at 1 mg/mL and was neutralized before use. Concentrations of antibiotics and extra carbon sources used for the treatment of *K. pneumoniae* are listed in the Supplementary Materials, Table S1. MHB medium was purchased from Bioyee (Beijing, China) and prepared as broth according to the manufacturer’s instructions. All strains were grown overnight in a 37 °C shaking incubator (200 rpm) prior to initiating the experiment.

### 4.2. Transcriptome Analysis

Transcriptome analysis of *K. pneumoniae* was conducted based on our previous method [40]. Bacterial cells were harvested and immediately treated with RNA protect Bacteria Reagent (QIAGEN Inc., Valencia, CA, USA) to minimize RNA degradation before harvest. Subsequently, the cells were collected and total RNA was extracted using the RNeasy Mini kit (QIAGEN Inc., Valencia, CA, USA). DNA was then digested with RNAse-free DNAseI (10 U/40 mg of total bacterial RNA) at 37 °C for 20 min. RNA was purified with RNeasy Mini column (QIAGEN Inc., Valencia, CA, USA). RNA quality was monitored via agarose gel electrophoresis and RNA quantity was measured via UV spectrophotometry. Then, cDNA was synthesized from 5 μg of total RNA in a 40 μL total reaction volume using a superscript III first-strand synthesis system (Invitrogen, Carlsbad, CA, USA). cDNA cleanup was carried out using Qiaquick PCR purification kit (QIAGEN Inc., Valencia, CA, USA). Next, sequencing was conducted at Hanyu Biotech Laboratory (Shanghai, China).

### 4.3. Next Generation Sequencing Data Analysis

Data analyses were conducted based on our previous pipeline [40]. Briefly, the global sequencing reads were checked for quality using FastQC, and the adapters were trimmed using trimmomatic [41]. The trimmed data for each group were aligned to our sequenced *K. pneumoniae* genome (GCA_015832035.1) using bowtie2. Then, Featurecount was applied to calculate the number of reads in each gene, and DESeq2 was used to identify the differential expressed genes [42].

### 4.4. Genetic Modifications

The backbone of expression vector pET-28a was used for overexpression of genes. Since the T7 promoter is unable to be used in *K. pneumoniae*, it was thereby replaced by the *tac* promoter according to our previous work [43]. Moreover, the chloramphenicol resistance gene from pCP20 was amplified and used to replace the kanamycin resistance gene because kanamycin was used for gene deletion. The modified plasmid ptac-control was used for the overexpression of genes in *K. pneumoniae*, and the genes were assembled into the vector using the Gibson Assembly Kit from NEB. Recombinant plasmids were electro-transformed into *K. pneumoniae* to generate the overexpressors. Gene deletion was conducted via an allelic exchange method with a pmob-sacB plasmid [44]. Briefly, 1000 bp of the upstream and downstream regions of the desired gene were amplified and then assembled with linear pmob-sacB plasmid using the Gibson assembly method. The recombinant plasmid was transformed into *E. coli* and kanamycin (10 μg/mL) was used to sustain the plasmid. After cultivation, bacteria were spread on an LB plate with kanamycin, and genomic insertion of the kanamycin resistance gene was identified via colony PCR. Next, recombinants were cultivated in an LB medium in the presence of 10% sucrose to eliminate the kanamycin resistance gene. A single colony after PCR verification of gene deletion was subjected to further study. Recombinant strains used in this work are listed in the Supplementary Materials, Table S1, and the primers used for genetic modification are listed in Table S2.

### 4.5. Determination of Minimum Inhibitory Concentration (MIC)

MICs were determined by use of a microplate assay to test antibiotic activity against recombinant *K. pneumoniae* according to the instructions of the Clinical and Laboratory Standards Institute (CLSI). The medium used in overnight culture and MIC assay were conducted in MHB broth containing chloramphenicol (100 μg/mL) and inducer IPTG (0.5 mM). First, OD_600_ of the overnight cultures was measured to maintain the same number of cells in each group (1.5 × 10^8^ cfu/mL), and cells were diluted 10,000-fold into a 96-well plate. Then, two-fold dilutions of each working solution were prepared, and the last cell in each row was set as a control. Plates were incubated at 37 °C for 12 h. The MIC assays were repeated independently three times.

### 4.6. Growth Curve and Fluorescence Intensity Assay

Strains were grown overnight in MHB medium containing chloramphenicol (100 μg/mL) and inducer IPTG (0.5 mM). After washing with fresh medium, the cells were diluted 10,000-fold into the same medium and then transferred into a 96-well plate for growth curve determination at 37 °C. The growth and fluorescence of each 100 μL culture in the plate was monitored via optical density at 600 nm (OD_600_) using a SpectraMax i3x (Molecular Devices, San Jose, CA, USA) plate reader at 37 °C. The experiment was independently carried out three times.

### 4.7. Time-Dependent Killing Experiments

Overnight cultures of each isolate were grown in MHB medium without IPTG at 37 °C and diluted 1:10 the following day in a fresh MHB liquid medium containing a final concentration of 0.5 mM IPTG and nearly 50 times the MICs of certain antibiotics. At each time point, the cells were centrifugally separated (8000× *g*, 5 min) from the medium containing antibiotics, and viable cell counts were determined via 10-fold serial dilution of cells in MHB agar and spot-plating 10 μL of each on plates. Colonies were counted after 12 h of growth at 37 °C. All the experiments were independently conducted in triplicate to calculate the standard error.

### 4.8. Gene Expression Analysis

In order to investigate the expression levels of *kpsrmfs* in different conditions, we constructed 2 reporter strains that use *egfp* as the reporter signal. First, we constructed a reporter strain on a plasmid. The native promoter *Pstr* of *kpsrmfs* was fused with the *egfp* gene, and they were co-integrated into the pET plasmid backbone without its native promoter. This plasmid was then transferred into the *K. pneumoniae* wt strain so that the copy number of the *Pstr* promoter was artificially increased. At the same time, we also constructed a mutant strain in which *kpsrmfs* was replaced by an *egfp* gene on the chromosome. The *egfp* gene together with the upstream and downstream fragments of *kpsrmfs* was assembled into pTOX6 plasmid and the resulting plasmid was transferred into *K. pneumoniae* to generate the mutant by two cycles of crossover [45]. To test the expression level of the *Pstr* promoter, antibiotics with 0.5 times the MICs were added to the medium to ensure cell growth. For carbon sources that can be used by *K. pneumoniae*, glucose, sucrose, glycerol, and lactose were individually supplied at the concentration of 5 g/L. After cultivation in a shake flask for 12 h, fluorescence was measured with excitation at 488 nm and emission at 520 nm. These experiments were biologically repeated in MHB medium three times.

### 4.9. Tetracycline Efflux and Accumulation Assay

Tetracycline efflux and accumulation were conducted according to references with modifications [27,43]. Briefly, in the efflux assay, overnight cultures of the cells harboring certain plasmids were diluted with NaCl-free LB medium (with 0.1 mM MgCl_2_ provided) to OD_600_ near 1.0. Then, 0.5 mM IPTG was added for 2 h. Next, 100 μg/mL of tetracycline was loaded and cultivated for another 1 h. When the balance accumulation of tetracycline was reached, the bacteria were centrifuged at 12,000 rpm for 3 min and the medium was replaced by 50 mM PBS buffer (pH 5.0). Aliquots of 0.1 mL were transferred to 0.2 mL microtubules and the assay was performed at 37 °C. The reaction was started when 5 g/L of glucose was provided, and samples were measured every 1.5 min. The efflux of tetracycline is presented in terms of fluorescence with excitation at 400 nm and emission at 520 nm. The inhibitor verapamil (50 μg/mL) or NaCl (0.25 M) was provided at 5 min to assess their effects on the efflux efficiency.

We also tested the intracellular tetracycline concentrations of the recombinant strains according to the references [14,46]. Briefly, recombinant strains were cultured in LB broth overnight with shaking. Cultures were diluted 100-fold in fresh MHB and then cultivated to the logarithmic phase, and then 0.5 mM of IPTG was provided and cultivated for 2 h. The cells were then harvested via centrifugation at 4 °C, washed twice with 50 mM PBS buffer (pH 7.0), and resuspended in the same buffer to an OD_600_ of 1. The cells were cultivated at 37 °C for 10 min, and then 100 μg/mL of tetracycline was added and cultivated for another 1 h. Subsequently, the samples were taken and divided evenly into two parts to measure cell dry weight and intracellular tetracycline concentration separately. To determine intracellular tetracycline concentration at a certain time point, the samples were taken and immediately diluted with 500 μL of cold PBS buffer. After centrifugation at 6000 rpm for 5 min, the cells were washed twice with cold PBS buffer and then resuspended in 0.1 M of glycerin hydrochloride (pH 3.0) and shaken at 37 °C for 5 h. After final centrifugation at 12,000 rpm for 10 min, the supernatants were filtered through a 0.22 μm membrane and used for tetracycline concentration analysis via the high-performance liquid chromatography (HPLC) method using a UV detector (SHIMADZU LC-16A, Suzhou, China). These experiments were conducted for three biological replicates and the standard errors were calculated.

### 4.10. Identification of Reactive Oxygen Species (ROS) Levels

ROS levels of the recombinant *K. pneumoniae* strains that express different genes were measured using the ROS assay kit S0033S from Beyotime (Beijing, China) according to the manufacturer’s instructions. Briefly, the recombinant strains were cultivated in MHB medium overnight without IPTG supplied. Next, the medium was diluted 10 times into fresh MHB medium with 0.5 mM of IPTG, and the ROS probe DCFH-DA was added to the final concentration of 10 μM. Then, the cells were cultivated at 37 °C without shaking for 3 h and the fluorescence was measured at a certain dilution level. Fluorescence was detected with an excitation of 488 nm and an emission of 525 nm. The experiments were conducted in biological triplicate.

### 4.11. Measurement of Catalase and Superoxide Dismutase Activities

Catalase and superoxide dismutase activities were determined using the Catalase assay kit S0051 and the Superoxide Assay Kit S0060 from Beyotime (Beijing, China) according to the manufacturer’s instructions. Cell cultures with arabinose induced were harvested via centrifugation after 12 h of cultivation. After washing with 50 mM of PBS buffer (pH 7.0) 2 times, the cells were disrupted in an ultra-sonic crusher with the proper amount of β-mercaptoethanol added, and the crude extract was used to conduct the enzyme activity assay. The total protein amount was estimated using the Coomassie Brilliant Blue Staining method with a standard curve from the estimation of Bovine Serum Albumin.

### 4.12. Statistical Analysis

Data of the growth curve were presented as mean values ± standard errors of mean (SD). Statistical significances are indicated by *p*-values analyzed via one-way ANOVA.

## 5. Conclusions

Efflux pumps play an important role in cell detoxification and are often up-regulated in the presence of antibiotics. Herein, we discovered the MFS efflux pump, KpsrMFS, which was down-regulated in different antibiotics and extra carbon sources, and its overexpression conferred halted cell growth. We investigated the role of *kpsrmfs* together with its adjacent genes. The results indicate a special regulon in regulating cell growth and endogenous ROS levels. Our work provides new insights into the role of efflux pumps in response to antibiotic stress.

## Figures and Tables

**Table 1:** Top 25 fluctuated genes in *K. pneumoniae* by determination of their standard deviations.

Number	Gene ID	Annotation	Gene Name	Standard Deviation
0	I5G01_02020	50S ribosomal protein L22	*rplV*	4.51
1	I5G01_04320	Type 3 fimbria major subunit MrkA	*mrkA*	4.83
2	I5G01_05265	S8 family peptidase	Non	4.32
3	I5G01_05895	Helix-turn-helix domain-containing protein	Non	4.33
4	I5G01_08000	Hypothetical protein	Non	4.79
5	I5G01_08515	Mannose-1-phosphate guanylyltransferase/mannose-6-phosphate isomerase	Non	4.65
6	I5G01_08520	O9 family phosphomannomutase RfbK1	*rfbK1*	4.88
7	I5G01_08570	Glycosyltransferase	Non	4.70
8	I5G01_08575	Hypothetical protein	Non	4.76
9	I5G01_08580	Glycosyltransferase family 2 protein	Non	4.52
10	I5G01_08835	Winged helix-turn-helix domain-containing protein	Non	4.96
11	I5G01_10545	Major outer membrane lipoprotein	Non	4.88
12	I5G01_10880	HAMP domain-containing histidine kinase CrrB	*ccrB*	4.50
13	I5G01_10885	Response regulator	Non	4.63
14	I5G01_10985	MFS transporter	*KpsrMFS*	4.33
15	I5G01_10995	Tautomerase family protein	Non	4.35
16	I5G01_11005	Chromate resistance protein	Non	4.40
17	I5G01_11075	DeoR/GlpR family DNA-binding transcription regulator	Non	4.46
18	I5G01_12185	LysE family translocator	Non	4.42
19	I5G01_15370	EAL domain-containing protein	Non	4.51
20	I5G01_22760	Polysaccharide pyruvyl transferase family protein	Non	4.84
21	I5G01_22765	Glycosyltransferase	Non	5.30
22	I5G01_22775	PAAR domain-containing protein	Non	4.41
23	I5G01_22800	Hypothetical protein	Non	4.32
24	I5G01_22810	AAA family ATPase	Non	4.50
25	I5G01_22825	N-6 DNA methylase	Non	4.39

**Table 2:** MIC data of the recombinant *K. pneumoniae* strains.

Strain	Kp-wt	KpΔ*kpsrmfs*	Kp-pET-backbone	Kp-pET-*Pstr*-*kpsrmfs*
MIC (μg/mL)				
Meropenem	0.0025	0.0025	0.00125	0.00125
Chloramphenicol	0.02	0.02	0.01	0.01
Ciprofloxacin	0.002	<0.0004	0.0004	0.0004
Tetracycline	0.25	0.03125	0.0625	0.125
Azithromycin	0.1	0.025	0.05	0.1
Rifampicin	1	1	0.5	0.5
Streptomycin	0.125	0.125	0.0625	0.0625
Hygromycin	0.0625	0.0625	0.0625	0.0625

**Table 3:** Adjacent genes of *kpsrmfs*.

Name	Annotation	Gene Name	Gene ID
U5	3-oxoacid CoA-transferase subunit B	Non	I5G01_10960
U4	CoA transferase subunit A	Non	I5G01_10965
U3	LysR family transcriptional regulator	Non	I5G01_10970
U2	Hypothetical protein	Non	I5G01_10975
U1	DUF535 family protein	Non	I5G01_10980
KpsrMFS	MFS transporter	*kpsrmfs*	I5G01_10985
D1	Helix-turn-helix transcriptional regulator	Non	I5G01_10990
D2	Tautomerase family protein	Non	I5G01_10995
D3	MFS transporter	Non	I5G01_11000
D4	Chromate resistance protein	Non	I5G01_11005
D5	Chromate efflux transporter	*chrA*	I5G01_11010

## Data Availability

Data of this study are available via email to the corresponding author.

## Supplementary Materials

The following supporting information can be downloaded at: https://www.mdpi.com/article/10.3390/ijms25031466/s1.

## Abbreviations

MFS	Major facilitator superfamily
ROS	Reactive oxygen species
ABC	ATP-binding cassette family
RND	Resistance/nodulation/cell division family
MATE	Multi-drug and toxic compound extrusion family
SMR	Small multi-drug resistance family
PACE	Proteobacterial antimicrobial compound efflux family
AbgT	p-Aminobenzoyl-glutamate transporter family
IPTG	Isopropyl β-D-1-thiogalactopyranoside
MHB	Mueller–Hinton Broth
LB	Luria–Bertani medium
MIC	Minimum inhibitory concentration
DCCM	Dynamical Cross-Correlation Matrix
CHL	Chloramphenicol

## References

[1] Paczosa M.K., Mecsas J. (2016). *Klebsiella pneumoniae*: Going on the Offense with a Strong Defense. Microbiol. Mol. Biol. Rev..

[2] Choby J.E., Howard-Anderson J., Weiss D.S. (2020). Hypervirulent *Klebsiella pneumoniae*—Clinical and Molecular Perspectives. J. Intern. Med..

[3] Russo T.A., Marr C.M. (2019). Hypervirulent *Klebsiella pneumoniae*. Clin. Microbiol. Rev..

[4] Navon-Venezia S., Kondratyeva K., Carattoli A. (2017). *Klebsiella pneumoniae*: A Major Worldwide Source and Shuttle for Antibiotic Resistance. FEMS Microbiol. Rev..

[5] Delmar J.A., Su C.-C., Yu E.W. (2014). Bacterial Multidrug Efflux Transporters. Annu. Rev. Biophys..

[6] Nolivos S., Cayron J., Dedieu A., Page A., Delolme F., Lesterlin C. (2019). Role of AcrAB-TolC Multidrug Efflux Pump in Drug-Resistance Acquisition by Plasmid Transfer. Science.

[7] Li J., Zhang H., Ning J., Sajid A., Cheng G., Yuan Z., Hao H. (2019). The Nature and Epidemiology of OqxAB, a Multidrug Efflux Pump. Antimicrob. Resist. Infect. Control.

[8] Ahmad I., Nawaz N., Dermani F.K., Kohlan A.K., Saidijam M., Patching S.G. (2018). Bacterial Multidrug Efflux Proteins: A Major Mechanism of Antimicrobial Resistance. Curr. Drug Targets.

[9] Teelucksingh T., Thompson L.K., Cox G. (2020). The Evolutionary Conservation of *Escherichia coli* Drug Efflux Pumps Supports Physiological Functions. J. Bacteriol..

[10] Rizzi A., Roy S., Bellenger J.-P., Beauregard P.B. (2019). Iron Homeostasis in *Bacillus subtilis* Requires Siderophore Production and Biofilm Formation. Appl. Environ. Microbiol..

[11] Alav I., Sutton J.M., Rahman K.M. (2018). Role of Bacterial Efflux Pumps in Biofilm Formation. J. Antimicrob. Chemother..

[12] Huang L., Wu C., Gao H., Xu C., Dai M., Huang L., Hao H., Wang X., Cheng G. (2022). Bacterial Multidrug Efflux Pumps at the Frontline of Antimicrobial Resistance: An Overview. Antibiotics.

[13] Li Y., Ge X. (2022). Molecular Dynamics Investigation of MFS Efflux Pump MdfA Reveals an Intermediate State between Its Inward and Outward Conformations. Int. J. Mol. Sci..

[14] Lv L., Wan M., Wang C., Gao X., Yang Q., Partridge S.R., Wang Y., Zong Z., Doi Y., Shen J. (2020). Emergence of a Plasmid-Encoded Resistance-Nodulation-Division Efflux Pump Conferring Resistance to Multiple Drugs, Including Tigecycline, in *Klebsiella pneumoniae*. mBio.

[15] Du D., van Veen H.W., Luisi B.F. (2015). Assembly and Operation of Bacterial Tripartite Multidrug Efflux Pumps. Trends Microbiol..

[16] Alcalde-Rico M., Hernando-Amado S., Blanco P., Martínez J.L. (2016). Multidrug Efflux Pumps at the Crossroad between Antibiotic Resistance and Bacterial Virulence. Front. Microbiol..

[17] Lamarche M.G., Déziel E. (2011). MexEF-OprN Efflux Pump Exports the Pseudomonas quinolone Signal (PQS) Precursor HHQ (4-Hydroxy-2-Heptylquinoline). PLoS ONE.

[18] Li Y. (2003). A New Member of the Tripartite Multidrug Efflux Pumps, MexVW-OprM, in *Pseudomonas aeruginosa*. J. Antimicrob. Chemother..

[19] Turner W.J., Dunlop M.J. (2015). Trade-Offs in Improving Biofuel Tolerance Using Combinations of Efflux Pumps. ACS Synth. Biol..

[20] Opperman T.J., Kwasny S.M., Kim H.-S., Nguyen S.T., Houseweart C., D’Souza S., Walker G.C., Peet N.P., Nikaido H., Bowlin T.L. (2014). Characterization of a Novel Pyranopyridine Inhibitor of the AcrAB Efflux Pump of *Escherichia coli*. Antimicrob. Agents Chemother..

[21] Srinivasan V.B., Rajamohan G. (2013). KpnEF, a New Member of the *Klebsiella pneumoniae* Cell Envelope Stress Response Regulon, Is an SMR-Type Efflux Pump Involved in Broad-Spectrum Antimicrobial Resistance. Antimicrob. Agents Chemother..

[22] Sun J., Deng Z., Yan A. (2014). Bacterial Multidrug Efflux Pumps: Mechanisms, Physiology and Pharmacological Exploitations. Biochem. Biophys. Res. Commun..

[23] Truong-Bolduc Q.C., Bolduc G.R., Okumura R., Celino B., Bevis J., Liao C.-H., Hooper D.C. (2011). Implication of the NorB Efflux Pump in the Adaptation of *Staphylococcus aureus* to Growth at Acid pH and in Resistance to Moxifloxacin. Antimicrob. Agents Chemother..

[24] Van Acker H., Coenye T. (2016). The Role of Efflux and Physiological Adaptation in Biofilm Tolerance and Resistance. J. Biol. Chem..

[25] Li Y., Cross T.S., Dörr T. (2022). Analysis of AcrB in *Klebsiella pneumoniae* Reveals Natural Variants Promoting Enhanced Multidrug Resistance. Res. Microbiol..

[26] Cross T., Ransegnola B., Shin J.-H., Weaver A., Fauntleroy K., VanNieuwenhze M.S., Westblade L.F., Dörr T. (2019). Spheroplast-Mediated Carbapenem Tolerance in Gram-negative Pathogens. Antimicrob. Agents Chemother..

[27] Truong-Bolduc Q.C., Wang Y., Hooper D.C. (2022). Role of *Staphylococcus aureus* Tet38 in Transport of Tetracycline and Its Regulation in a Salt Stress Environment. J. Bacteriol..

[28] Skulj M., Okrslar V., Jalen S., Jevsevar S., Slanc P., Strukelj B., Menart V. (2008). Improved Determination of Plasmid Copy Number Using Quantitative Real-Time PCR for Monitoring Fermentation Processes. Microb. Cell Factories.

[29] Li Y., Ge X.-Z., Tian P.-F. (2017). Production of 1,3-Propanediol from Glycerol Using a New Isolate *Klebsiella* sp. AA405 Carrying Low Levels of Virulence Factors. Biotechnol. Biotechnol. Equip..

[30] Wu C.-J., Chen Y., Li L.-H., Wu C.-M., Lin Y.-T., Ma C.-H., Yang T.-C. (2022). Roles of SmeYZ, SbiAB, and SmeDEF Efflux Systems in Iron Homeostasis of *Stenotrophomonas maltophilia*. Microbiol. Spectr..

[31] Thomas E., Roman E., Claypool S., Manzoor N., Pla J., Panwar S.L. (2013). Mitochondria Influence *CDR1* Efflux Pump Activity, Hog1-Mediated Oxidative Stress Pathway, Iron Homeostasis, and Ergosterol Levels in *Candida albicans*. Antimicrob. Agents Chemother..

[32] Vecchione J.J., Alexander B., Sello J.K. (2009). Two Distinct Major Facilitator Superfamily Drug Efflux Pumps Mediate Chloramphenicol Resistance in *Streptomyces coelicolor*. Antimicrob. Agents Chemother..

[33] Lamarche M.G., Wanner B.L., Crépin S., Harel J. (2008). The Phosphate Regulon and Bacterial Virulence: A Regulatory Network Connecting Phosphate Homeostasis and Pathogenesis. FEMS Microbiol. Rev..

[34] Wood L.F., Ohman D.E. (2012). Identification of Genes in the σ ^22^ Regulon of *Pseudomonas aeruginosa* Required for Cell Envelope Homeostasis in Either the Planktonic or the Sessile Mode of Growth. mBio.

[35] Skrt M., Jamnik P., Poklar Ulrih N. (2018). Antioxidative Activity of Methanolic and Water Extracts from the Hyperthermophilic Archaeon *Aeropyrum pernix* K1. Acta Chim. Slov..

[36] Bonds A.C., Sampson N.S. (2018). More than Cholesterol Catabolism: Regulatory Vulnerabilities in *Mycobacterium tuberculosis*. Curr. Opin. Chem. Biol..

[37] Aggett R., Mallette E., Gilbert S.E., Vachon M.A., Schroeter K.L., Kimber M.S., Seah S.Y.K. (2019). The Steroid Side-Chain–Cleaving Aldolase Ltp2–ChsH2DUF35 Is a Thiolase Superfamily Member with a Radically Repurposed Active Site. J. Biol. Chem..

[38] Kim H., Choe J. (2013). The X-Ray Crystal Structure of PA1374 from *Pseudomonas aeruginosa*, a Putative Oxidative-Stress Sensing Transcriptional Regulator. Biochem. Biophys. Res. Commun..

[39] Newberry K.J., Fuangthong M., Panmanee W., Mongkolsuk S., Brennan R.G. (2007). Structural Mechanism of Organic Hydroperoxide Induction of the Transcription Regulator OhrR. Mol. Cell.

[40] Li Y., He R., Ge X. (2022). Glycerol Promotes Biomass Accumulation of *Klebsiella pneumoniae* by Activating *dha* Regulon. Process Biochem..

[41] Bolger A.M., Lohse M., Usadel B. (2014). Trimmomatic: A Flexible Trimmer for Illumina Sequence Data. Bioinformatics.

[42] Varet H., Brillet-Guéguen L., Coppée J.-Y., Dillies M.-A. (2016). SARTools: A DESeq2- and EdgeR-Based R Pipeline for Comprehensive Differential Analysis of RNA-Seq Data. PLoS ONE.

[43] Li Y., Ge X. (2022). Discovering Interrelated Natural Mutations of Efflux Pump KmrA from *Klebsiella pneumoniae* That Confer Increased Multidrug Resistance. Protein Sci..

[44] Schäfer A., Tauch A., Jäger W., Kalinowski J., Thierbach G., Pühler A. (1994). Small Mobilizable Multi-Purpose Cloning Vectors Derived from the *Escherichia coli* Plasmids pK18 and pK19: Selection of Defined Deletions in the Chromosome of Corynebacterium Glutamicum. Gene.

[45] Lazarus J.E., Warr A.R., Kuehl C.J., Giorgio R.T., Davis B.M., Waldor M.K. (2019). A New Suite of Allelic-Exchange Vectors for the Scarless Modification of Proteobacterial Genomes. Appl. Environ. Microbiol..

[46] Mortimer P.G.S., Piddock L.J.V. (1991). A Comparison of Methods Used for Measuring the Accumulation of Quinolones by Enterobacteriaceae, *Pseudomonas aeruginosa* and *Staphylococcus aureus*. J. Antimicrob. Chemother..

