# Supplementary material for: Identification of the Major Facilitator Superfamily Efflux Pump KpsrMFS in Klebsiella pneumoniae That Is Down-Regulated in the Presence of Multi-Stress Factors
Source: Int J Mol Sci. 2024 Jan 25;25(3):1466. doi: 10.3390/ijms25031466 (PMC10855805; doi:10.3390/ijms25031466)
Supplement: Supplementary file 1 [file ijms-25-01466-s001.zip › ijms-2800538-supplementary.pdf]

Supplemental Figure 1. SDS-PAGE analysis of KpsrMFS overexpression in *K. pneumoniae*. Overnight culture of Kp-*tac*-*kpsrmfs* was incubated at 37 ºC for another 3 h with IPTG induced (Line 2), and the same strain without induction (Line 1) was set as the control. M: protein marker WJ103 from epizyme co., ltd.

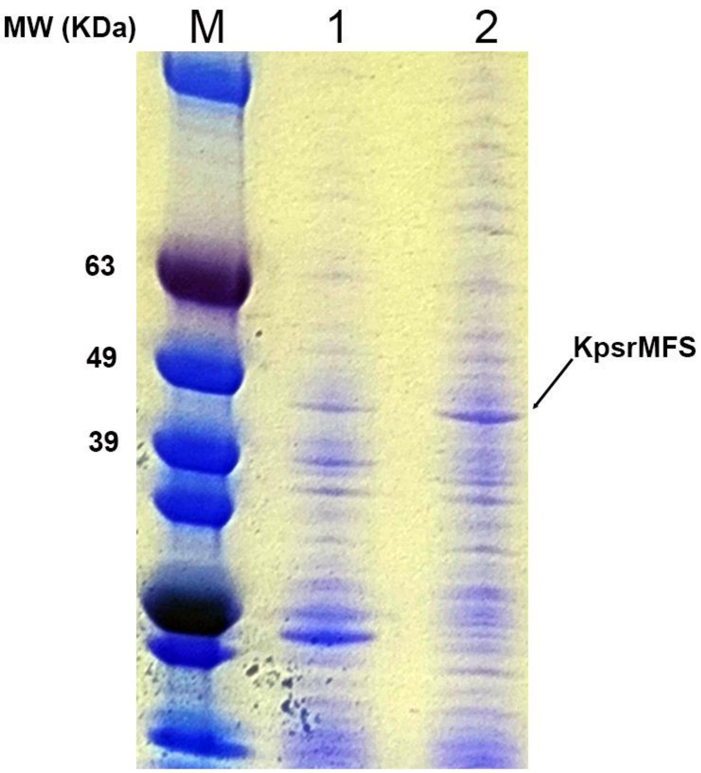


Supplemental table 1. Strains of *Klebsiella pneumoniae* used in this study.

| Strain name | Genopyte/Description | Source | Growth medium/Antibiotics |
|---|---|---|---|
| Wt | Wild type strain | Isolated from environment, Accession number ASM1583203v1 | MHB/None |
| Kp-*tac*-control | pET-28a derived plasmid pCHL-*tac* (kanamycin resistance gene and T7 promoter were replaced by chloramphenicol resistance | This work | MHB/cloramphenicol |

| | | | |
|---|---|---|---|
| | gene and *tac* promoter, respectively) | | |
| Kp-*tac-kpsrmfs* | Overexpression of *kpsrmfs* | This work | MHB/chloramphenicol |
| KpΔ*kpsrmfs* | Δ*kpsrmfs* mutant | This work | MHB/None |
| Kp-pET-backbone | pET-28a plasmid with T7 promoter deleted | This work | MHB/kanamycin |
| Kp-pET-*Pstr-kpsrmfs* | pET-backbone ligated with *kpsrmfs* together with its native promoter *Pstr* | This work | MHB/kanamycin |
| Kp-*Pstr-egfp* | pCHL plasmid (without *tac* promoter) with *Pstr* promoter and *egpf* reporter gene | This work | MHB/kanamycin |
| KpΔ*kpsrmfs*::*egfp* | Δ*kpsrmfs* replaced by *egfp* | This work | MHB/None |
| Kp-*tac-U1* | Overexpression of DUF535 family protein (I5G01_10980) | This work | MHB/chloramphenicol |
| Kp-*tac-U2* | Overexpression of Hypothetical protein (I5G01_10975) | This work | MHB/chloramphenicol |
| Kp-*tac-U3* | Overexpression of LysR family transcriptional regulator (I5G01_10970) | This work | MHB/chloramphenicol |
| Kp-*tac-U4* | Overexpression of CoA transferase subunit A(I5G01_10965) | This work | MHB/chloramphenicol |
| Kp-*tac-U5* | Overexpression of 3-oxoacid CoA-transferase subunit B (I5G01_10960) | This work | MHB/chloramphenicol |
| Kp-*tac-D1* | Overexpression of Helix-turn-helix transcriptional regulator (I5G01_10990) | This work | MHB/chloramphenicol |

| | | | |
|---|---|---|---|
| Kp-*tac-D2* | Overexpression of Tautomerase family protein (I5G01_10995) | This work | MHB/chloramphenicol |
| Kp-*tac-D3* | Overexpression of MFS transporter (I5G01_11000) | This work | MHB/chloramphenicol |
| Kp-*tac-D4* | Overexpression of Chromate resistance protein (I5G01_11005) | This work | MHB/chloramphenicol |
| Kp-*tac-D5* | Overexpression of Chromate efflux transporter (I5G01_11010) | This work | MHB/chloramphenicol |
| Kp-*tac-kpsrmfs-pBAD* | pCHL-*tac-kpsrmfs* and pBAD18-kan double vector | This work | MHB/chloramphenicol/kanamycin |
| Kp-*tac-kpsrmfs-pBAD-katG* | pCHL-*tac-kpsrmfs* and pBAD18-kan-*katG* double vector | This work | MHB/chloramphenicol/kanamycin |
| Kp-*tac-kpsrmfs-pBAD-sodB* | pCHL-*tac-kpsrmfs* and pBAD18-kan-*sodB* double vector | This work | MHB/chloramphenicol/kanamycin |

Supplemental Table 2. Primers used in this work.

| Name | Sequence (5’-3’) | Description |
|---|---|---|
| U-1-F | tagaaataattttgtttaactttaagaaggagatatacccatggaacatactgttgcagg | *U1* PCR forward |
| U-1-R | ggccccaaggggttatgctagttattgctcagcggtgcccttaatttacccgcgcaggca | *U1* PCR reverse |
| U-2-F | tagaaataattttgtttaactttaagaaggagatatacccatggaaattgatctcgacaa | *U2* PCR forward |
| U-2-R | ggccccaaggggttatgctagttattgctcagcggtgccctcagattttacaggcgtcgc | *U2* PCR reverse |
| U-3-F | tagaaataattttgtttaactttaagaaggagatatacccatgagaatgagtgtcaaaca | *U3* PCR forward |
| U-3-R | ggccccaaggggttatgctagttattgctcagcggtgccctcaggtgggtaaaaaccctt | *U3* PCR reverse |

| | | |
|---|---|---|
| U-4-F | tagaaataattttgtttaactttaagaaggagatatacccatggcaggactggataaacg | *U4* PCR forward |
| U-4-R | ggccccaaggggttatgctagttattgctcagcggtgccctcagtgccctccggcgcgca | *U4* PCR reverse |
| U-5-F | tagaaataattttgtttaactttaagaaggagatatacccatgctgacccgtgaacaaat | *U5* PCR forward |
| U-5-R | ggccccaaggggttatgctagttattgctcagcggtgcccttaactgaagcgcatttcgc | *U5* PCR reverse |
| D1-F | tagaaataattttgtttaactttaagaaggagatatacccatgtcaaaaaataaatcact | *D1* PCR forward |
| D1-R | ggccccaaggggttatgctagttattgctcagcggtgcccttagcgttcgtctgccttga | *D1* PCR reverse |
| D2-F | tagaaataattttgtttaactttaagaaggagatatacccatgccatttgttaacgtgca | *D2* PCR forward |
| D2-R | ggccccaaggggttatgctagttattgctcagcggtgcccttattccggggatccgtcgc | *D2* PCR reverse |
| D3-F | tagaaataattttgtttaactttaagaaggagatatacccatgtctacagaacacatgct | *D3* PCR forward |
| D3-R | ggccccaaggggttatgctagttattgctcagcggtgcccctaagcgcgggatggggata | *D3* PCR reverse |
| D4-F | tagaaataattttgtttaactttaagaaggagatatacccatgacaatgcatctcttgat | *D4* PCR forward |
| D4-R | ggccccaaggggttatgctagttattgctcagcggtgccctcactctgcggaagaacgac | *D4* PCR reverse |
| D5-F | tagaaataattttgtttaactttaagaaggagatatacccatgagcaaaacggtcgttct | *D5* PCR forward |
| D5-R | ggccccaaggggttatgctagttattgctcagcggtgccctcatcctgcgccggacagtc | *D5* PCR reverse |
| pCHL-rev-F | gggtatatctccttcttaaagttaaacaaaattatttcta | pCHL backbone PCR forward |
| pCHL-rev-R | gggcaccgctgagcaataactagcataaccccttggggcc | pCHL backbone PCR reverse |
| P-*egfp*-F | gggcagtgagcgcaacgcaattaattagcgccgctacagaactttcgtac | *egfp* gene PCR reverse |
| P-*egfp*-R | ttggcgggtgtcggggctggcttaattacttgtacagctcgtccatgccg | *egfp* gene PCR reverse |
| *Pstr*-F | atcccgcgaaatcgatcccgcgaaatagcgccgctacagaactttcgtac | Native promoter *Pstr* PCR forward |

| | | |
|---|---|---|
| *Pstr*-R | ccagtgaatccgtaatcatggtcattttctacctttagcatgcgcgatct | Native promoter *Pstr* PCR reverse |
| Pdel-1F | ccaagcttgcatgcctgcaggtcgactctagaggatcccctagcgccgctacagaacttt | Upstream fragment of *kpsrmfs* forward |
| Pdel-1R | gatgcatcgccagtaaccgtatttgcggccagcagcaagcgcttatgccg | Upstream fragment of *kpsrmfs* reverse |
| Pdel-2F | cggcataagcgcttgctgctggccgcaaatacggttactggcgatgcatc | Downstream fragment of *kpsrmfs* forward |
| Pdel-2R | aacagctatgaccatgattacgaattcgagctcggtacccttactgcccgctctcgccga | Downstream fragment of *kpsrmfs* reverse |
| Pdel-check-F | tcgcggcataagcgcttgctgctgg | *kpsrmfs* mutation check forward |
| Pdel-check-R | ccagcaggatgcatcgccagtaacc | *kpsrmfs* mutation check reverse |
| *kpsrmfs*-F | gcgtccggcgtagaggatcgagatctcgatcccgcgaaattagcgccgctacagaacttt | *kpsrmfs* PCR forward |
| *kpsrmfs*-R | agcggtggcagcagccaactcagcttcctttcgggctttgtttctacctttagcatgcgc | *kpsrmfs* PCR reverse |
| Cass-F | gcgtccggcgtagaggatcgagatctcgatcccgcgaaattagcgccgctacagaacttt | Cassette *Pstr-kpsrmfs* PCR forward |
| Cass-R | agcggtggcagcagccaactcagcttcctttcgggctttgttactgcccgctctcgccga | Cassette *Pstr-kpsrmfs* PCR reverse |
| *egfp*-U-F | cttcggcggggtttttcgctgatcacgtacgatgatctggcccctcttctgcgtcaga | Upstream of *kpsrmfs* PCR forward |
| *egfp*-U-R | acagctcctcgcccttgctcaccattttctacctttagcatgcgcgatct | Upstream of *kpsrmfs* PCR reverse |
| *egfp*-D-F | cggcatggacgagctgtacaagtaacctacggcacagcgacctggggaga | Downstream of *kpsrmfs* PCR forward |

| | | |
|---|---|---|
| *egfp*-D-R | gggccctgtacaccatgtgcaccggttcgaagattcttaatcaatggaacatactgttg | Downstream of *kpsrmfs* PCR reverse |
| *egfp*-del-F | agatcgcgcatgctaaaggtagaaaatggtgagcaagggcgaggagctgt | egfp PCR forward |
| *egfp*-del-R | tctccccaggtcgctgtgccgtaggttacttgtacagctcgtccatgccg | egfp PCR reverse |
| pBAD-Gib-F | ctcggtacccggggatcctctagagtcgacctgc | pBAD18a vector amplification without ampicillin resistance gene forward |
| pBAD-Gib-R | ctcgaattcgctagcccaaaaaaacgggtatggag | pBAD18a vector amplification without ampicillin resistance gene reverse |
| *kana*-F | ctgacggatggcctttttgcgtttctacaaactctttgatcttttctacggggtctgac | Kanamycin resistance gene PCR forward |
| *kana*-R | atctaaagtatatatgagtaaacttggtctgacagttagaaaaactcatcgagcatcaa | Kanamycin resistance gene PCR reverse |
| *KatG*-F | ctccatacccgtttttttgggctagcgaattcgagatgagcacgtctaacgacccatcc | *katG* PCR forward |
| *KatG*-R | tgcaggtcgactctagaggatccccgggtaccgagttacaggtcgaagcggtcgaggtt | *katG* PCR reverse |
| *SodB*-F | ctccatacccgtttttttgggctagcgaattcgagatgtcgtttgaattacctgcatta | *sodB* PCR forward |
| *SodB*-R | tgcaggtcgactctagaggatccccgggtaccgagttatgccgcgaggttagccgcaac | *sodB* PCR reverse |
